# Contamination of fungal genomes of Onygenaceae (Phylum Ascomycota) in public databases: incidence, detection, and impact

**DOI:** 10.1186/s12864-025-12223-3

**Published:** 2025-11-19

**Authors:** Alan Omar Granados-Casas, Ana Fernández-Bravo, Alberto Miguel Stchigel, José Francisco Cano-Lira

**Affiliations:** Faculty of Medicine, Rovira i Virgili University, Mycology Unit, C/Sant Llorenç, 21, Reus, Tarragona Province 43201 Spain

**Keywords:** *Ascomycota*, Contamination, Fungi, Onygenales, Whole genome sequencing

## Abstract

Genomic datasets often contain unwanted, foreign, or erroneous nucleotide sequences that do not belong to the organism under study. Such contamination can significantly compromise genome analyses, reducing the accuracy and reliability of the results. Despite its potential impact, few studies have addressed the contamination of fungal genomes by exogenous sequences. Here, we analyzed eleven publicly available genomes of fungi from the family Onygenaceae, retrieved from the National Center for Biotechnology Information (NCBI) database. A comprehensive quality assessment was performed, evaluating genome completeness, contiguity, and contamination levels. Genomes with lower statistical quality and putatively contaminated were selected for further improvement. To enhance assembly quality, we built a custom Kraken 2 database including four high-quality genomes of closely related fungal taxa. After filtering, we reassessed the genomes to compare contiguity, completeness, and contamination levels before and after the process. Furthermore, structural and functional annotation was conducted to evaluate changes in predicted proteins, protein families and domains. Additionally, Average nucleotide identity and phylogenetic analyses were performed to further assess the impact of the filtering. Four genomes showed low-quality statistics and contamination levels between 5 and 12%, mainly of bacteria origin. After removing the contaminated regions, assembly quality metrics improved, and contamination level dropped below 3% in all cases. Functional annotation of the filtered assemblies revealed a reduction in bacteria-associated protein families. Our results demonstrate the presence of contamination in publicly available Onygenaceae fungal genomes and highlight its potential to bias downstream analyses. We emphasize the importance of contamination screening and removal to ensure reliable genomic data for fungal research.

**Supplementary Information:**

The online version contains supplementary material available at 10.1186/s12864-025-12223-3.

## Importance

The presence of contaminating sequences in publicly available genomes presents a significant challenge. To address this issue, we identified and removed contaminant sequences in four fungal genomes belonging to different species of the family Onygenaceae. Our findings demonstrate that the use of a database of high-quality genomes closely related to the target genome can be effectively used to filter out potential contaminants. Furthermore, the results highlight the critical importance of rigorous quality control measures to ensure the accuracy and integrity of genomic data in molecular biology and genomics.

## Introduction

Technical advances in high-throughput sequencing and the resulting reduction in sequencing costs have led to a surge in the number of genomes available in public databases. These repositories of genetic data have enriched our comprehension of genome structure and evolution, population dynamics, and the rise of antifungal or antibiotic resistance mechanisms [1–7]. Nevertheless, this wealth of information comes with its own set of challenges, one of which is contaminant sequences.

According to the National Center for Biotechnology Information (NCBI), “A contaminated sequence is one that does not faithfully represent the genetic information from the biological source organism/organelle because it contains one or more sequence segments of foreign origin" (Contamination in sequence databases, NCBI, https://www.ncbi.nlm.nih.gov/tools/vecscreen/contam/#:~:text=(FCS)%20yourself!-,Definition,sequence%20segments%20of%20foreign%20origin). Contamination of the genetic material can occur at different stages of the sequencing process. For instance, the microbial strain may be contaminated during collection or while being stored in culture. Additionally, plastic consumables, laboratory equipment, or reagents/kits may be contaminated with foreign genetic material. Environmental contamination during sequencing may also result in the incorporation of exogenous DNA [8–10].

One of the main challenges of working with contaminated genomes is to detect and remove contamination without altering the genuine genetic content of the organism of interest. Consequently, bioinformatics tools have been developed to identify contaminants in both raw reads and assembled genomes. These tools can be classified according to the type of data they can analyze: Tools designed to analyze prokaryotic genomes, e.g., GUNC, CheckM, CLARK, and CONSULT [11–14]; tools designed to analyze eukaryotic genomes, such as EukCC [15]; and tools applicable to both domains, e.g., PhylOligo, BlobToolKit, Forty-Two, Kraken 2, and FCS-GX [16–20]. Notably, the latter two can be conveniently run on public Galaxy servers (public web access: http://usegalaxy.org), enabling their use without requiring local installation.

Kraken 2 is a taxonomic classifier that analyzes k-mers in a query sequence and uses this information to search a database. The database associates k-mers with the lowest common ancestor (LCA) of all genomes known to possess the specific k-mer. It offers several advantages over other contamination detection tools, including user-friendly installation and execution, the ability to detect cross-domain contamination, and precise estimation of contamination levels [21].

Several studies have reported contaminating sequences in public genome repositories [22–25]. Longo et al. (2011) identified significant contamination with human DNA across a range of genomes, including protists (e.g., *Chlamydomonas reinhardtii*), bacteria (*Bacillus cereus*), plants (*Zea mays*), and animals such as bird (*Gallus gallus*), and fish (*Danio rerio*). Subsequently, Merchant et al. [26] identified bacterial sequences within a *Bos taurus* genome, as well as sequences originating from sheep and cow as contaminants in a putatively complete genome of the human sexually transmitted bacterium *Neisseria gonorrhoeae*. Mukherjee et al. [27] reported over 1,000 publicly available genomes contaminated with PhiX sequences, a common quality control element in Illumina sequencing. In addition, Kryukov and Imanishi [25] observed evidence of human DNA contamination in genomes of non-primate mammals, non-mammalian vertebrates, non-vertebrate eukaryotes, and prokaryotes. Finally, Francois et al. [28] analyzed 46 arthropod reference genomes and identified eleven genomes with and identified eleven with minor contamination, while four showed substantial levels of contaminant sequences. Nonetheless, only a limited number of studies have assessed contamination specifically in fungal genomes [20, 29–32].

The order Onygenales encompasses fungi of significant clinical importance, including thermally dimorphic systemic pathogens (e.g. species of the genera *Blastomyces*, *Coccidioides,* and *Emergomyces*), dermatophytes (*Epidermophyton*, *Microsporum*, *Nannizzia* and *Trichophyton*), opportunistic fungal pathogens (*Emmonsia*, *Malbranchea* and *Spiromastigoides*), and saprobic, non-pathogenic species capable of degrading keratinous substrates, thereby contributing to their recycling [33–35]. Within this order, there is a limited but growing availability of reference genomes. Therefore, the use of contaminated genomes in downstream analyses, such as structural or functional annotation, comparative genomics, or phylogenomics, may compromise the accuracy of the results. For this reason, the objective of this study was to assess the presence of contaminant sequences in a selected set of publicly available reference genomes of fungi from the family Onygenaceae (orderOnygenales) in the NCBI database, improve their quality through decontamination, and to demonstrate the potential impact of using contaminated data on downstream analyses. 

## Materials and method

### Genomic data

Eleven reference genomes were selected from among the 112 Onygenaceae genomes available from the NCBI Genome Database (https://www.ncbi.nlm.nih.gov/genome/?term=Onygenaceae; accessed April 2023) (*Amauroascus* [*Am*.] niger UAMH 3544 (GCA_001430945.1), *Amauroascus* *verrucosus* UAMH 3576 (GCA_001430935.1), *Aphanoascus* [*Ap*.] *verrucosus* IHEM 4434 (GCA_014839905.1), *Brunneospora* [*Br*.] *queenslandica* (asexual morph *Chrysosporium queenslandicum*) CBS 280.77 (GCA_001430955.1), *Byssoonygena* [*By*.] ceratinophila UAMH 5669 (GCA_001430925.1), *Chrysosporium* [*Ch*.] *keratinophilum* CBS 104.62 (GCA_029850275.1), *Coccidioides* [*C*.] immitis RS (GCA_000149335.2), *Coccidioides posadasii* C735 delta SOWgp (GCA_000151335.1), *Nannizziopsis* [*Na*.] *barbatae* USC001 (GCA_014964245.1), *Ophidiomyces* [*Op*.] *ophidiicola* MYCO-ARIZ (GCA_002167195.1) and *Uncinocarpus* [*U*.] *reesii* UAMH 1704) (GCA_000003515.2) (Supplementary Table 1).

### Quality and completeness analyses

**Fig. 1:**
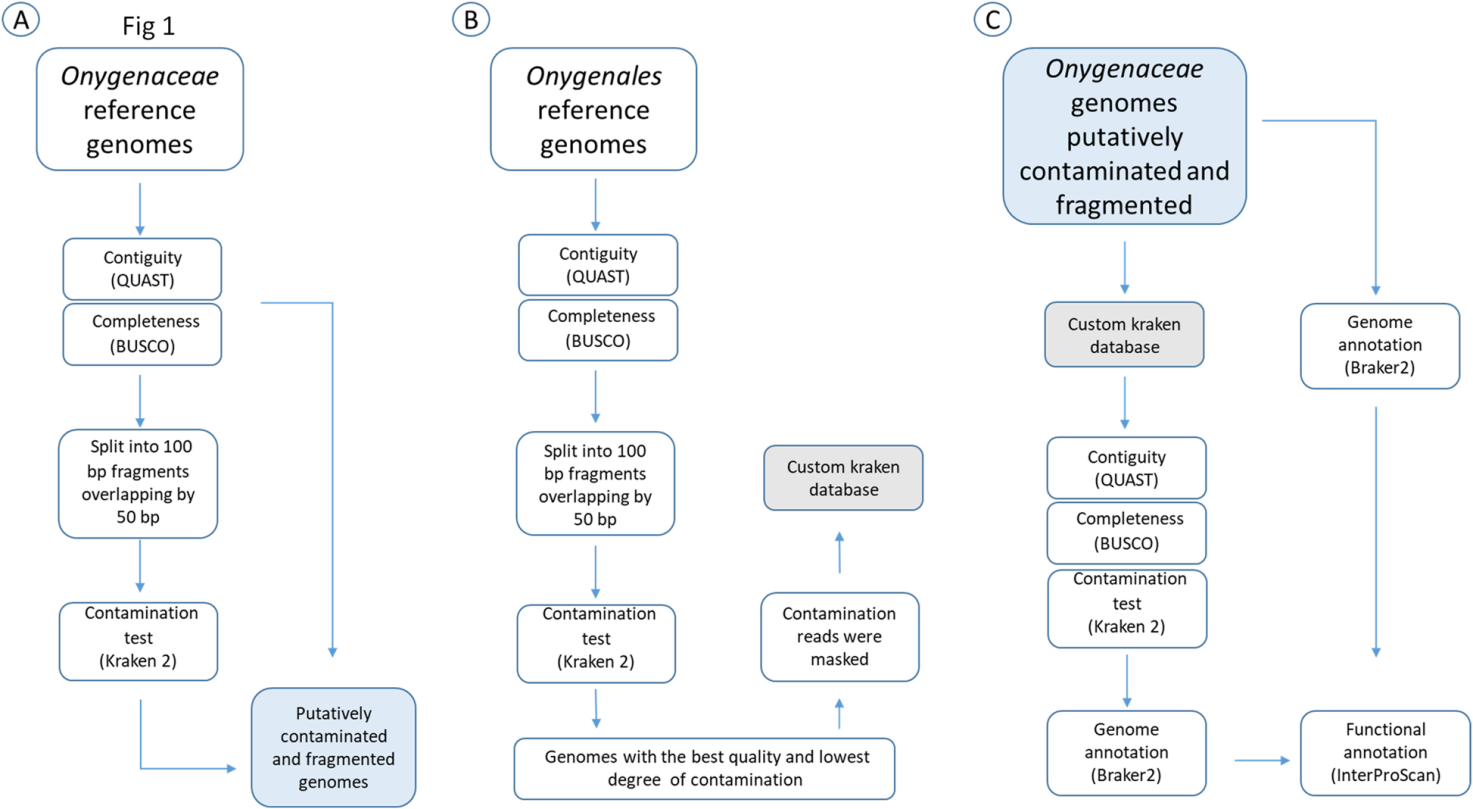
Workflow for improving the quality of Onygenaceae genomes. **A** Evaluation of quality, completeness and contamination in the original Onygenaceae genomes. **B** Methodology for selecting the genomes included in the custom database. **C** Assessment of the putatively contaminated, and the filtered genomes

Quality and completeness analyses of the genome assemblies were performed using the tools Quality Assessment Tool for Genome Assemblies (QUAST) v5.1.0 [36] and Benchmarking Universal Single-Copy Orthologs (BUSCO) v5.4.3 [37] (Fig. 1A). Specifically, QUAST was employed to evaluate the number of contigs, N50, N90, and the number of N's. The Auto-Select Lineage option was chosen to run BUSCO on the generic lineage datasets of *Archaea*, *Bacteria*, and *Eukarya*. The proportions of complete, fragmented, and missing BUSCOs genes were used to assess completeness.

### Contamination identification and removal

To assess potential contamination, the downloaded Onygenaceae genomes were fragmented into 100 bp reads overlapping by 50 bp using pyfasta [38]. These fragments were analyzed with Kraken 2 v2.0.8 [17] against the Standard Kraken 2 database (StandardDB), which contains Refseq sequences from archaeal, bacterial, viral, plasmid, human, and UniVec_core (Fig. 1A). To minimize false positives, sequences flagged as contaminants were further validated using BLAST. Genomes with low N50 values (< 300,000 bp), a high number of contigs (> 1,000) and L50 values (> 40), and a contamination percentage (≥ 5%) were selected for further improvement (Fig. 1A, Table 1).

**Table 1:** Statistics of reference Onygenaceae genomes

Species (Strain)		*Amauroascus niger* (UAMH 3544)	*Amauroascus verrucosus* (UAMH 3576)	*Aphanoascus verrucosus* (IHEM 4434)	*Brunneospora queenslandica* (CBS 280.77)	*Byssoonygena ceratinophila* (UAMH 5669)	*Chrysosoporium keratinophilum* (CBS 104.62)	*Coccidioides immitis* (RS)	*Coccidioides posadasii* (C735 delta SOWgp)	*Nannizziopsis barbatae* (USC001)	*Ophidiomyces ophidiicola* (MYCO-ARIZ)	*Uncinocarpus reesii* (UAMH 1704)
**Sequencing platform**		Illumina	Illumina	Illumina	Illumina	Illumina	Illumina + Pacbio	Sanger shotgun	Sanger shotgun	Illumina + Oxford Nanopore	Roche 454	Sanger shotgun
**Assembly tool**		SOAPdenovo	SOAPdenovo	SOAPdenovo	SOAPdenovo	SOAPdenovo	MaSuRCA	The Arachne package	The Arachne package	MaSuRCA	Newbler	The Arachne package
Assembly statistics												
**# Contigs**		3,481	3,075	211	2,724	4,851	25	7	55	11	116	45
**Total length (bp)**		36,718,296	30,376,016	23,059,040	32,335,957	27,454,949	25,439,844	29,016,019	27,013,412	31,543,341	21,970,319	22,349,738
**Largest contig (bp)**		564,389	836,833	894,230	979,930	643,678	5,001,415	8,482,323	5,398,309	9,294,961	1,803,704	7,891,746
**G + C content (%)**		50.09	49.26	49.59	53.15	48.44	49.09	45.96	46.59	40.36	47.64	48.66
**N50 (bp)**		98,932	217,754	431,853	173,791	103,459	2,037,736	4,323,945	2,376,830	6,192,128	506,472	5,232,914
**N90 (bp)**		3,899	3,183	132,993	4,367	1,404	460,884	3,458,857	974,251	2,386,870	122,144	2,507,206
**L50**		86	40	17	47	67	4	3	4	3	12	2
**L90**		1,178	784	52	798	1,651	14	6	11	5	42	5
**# N's per 100 kbp**		5,647.86	8,888.54	10.51	6,001.89	6,870.54	10.06	1.38	0.12	0.00	1.81	812.87
BUSCO statistics												
***Onygenales*_odb10**	C* (%)	96.0	96.7	96.3	96.3	94.1	96.0	96.8	96.8	94.5	94.8	93.0
	F*(%)	0.8	0.7	0.7	0.9	1.9	0.6	1.0	1.0	1.0	1.0	3.5
	M* (%)	3.2	2.6	3.0	2.8	4.0	3.4	2.2	2.2	4.5	4.2	3.5
	**n**	4,862	4,862	4,862	4,862	4,862	4,862	4,862	4,862	4,862	4,862	4,862
***Archaea*_odb10**		C:28.9%,n:194	C:19.6%,n:194	C:18.0%,n:194	C:29.9%,n:194	C:19.0%,n:194	C:19.6%,n:194	C:15.9%,n:194	C:18.0%,n:194	C:17.5%,n:194	C:18.0%,n:194	C:17.0%,n:194
***Bacteria*_odb10**		C:45.1%,n:124	C:16.1%,n:124	C:12.9%,n:124	C:30.6%,n:124	C:13.7%,n:124	C:10.5%,n:124	C:9.7%,n:124	C:8.9%,n:124	C:10.5%,n:124	C:11.3%,n:124	C:8.1%,n:124
***Eukaryota*_odb10**		C:98.1%,n:255	C:97.7%,n:255	C:99.2%,n:255	C:99.2%,n:255	C:96.5%,n:255	C:98.4%,n:255	C:97.3%,n:255	C:97.6%,n:255	C:98.4%,n:255	C:99.2%,n:255	C:93.7%,n:255
Percentage of fragments covered for each domain in the analyzed genomes obtained by Kraken 2 with StandardDB												
**Viruses**		0.02	0.01	0.01	0.01	0.02	0.01	0.02	0.01	0.04	0.01	0.01
**Archaea**		0.06	0.04	0.04	0.07	0.04	0.05	0.03	0.04	0.10	0.04	0.04
**Bacteria**		11.27	7.07	1.20	12.05	4.98	1.44	1.25	1.22	2.92	1.64	1.40
**Eukaryota**		1.12	0.83	0.28	0.41	0.58	0.39	1.78	1.86	2.25	0.99	0.38
** Total**		12.47	7.95	1.53	12.54	5.61	1.89	3.08	3.13	5.38	2.70	1.83
Summary of statistics from NCBI FCS results												
**Total sequences**		1,667	2,273	55	1,976	2,599	2	0	0	0	0	0
**Main contaminant type**		Betaproteobacteria	Alphaproteobacteria	Plants	Betaproteobacteria	Betaproteobacteria	Bacillota	-	-	-	-	-

C*, Complete BUSCOs; F*, Fragmented BUSCOs; M*, Missing BUSCOs; n, Total BUSCO groups searched

### Database creation

**Fig. 2:**
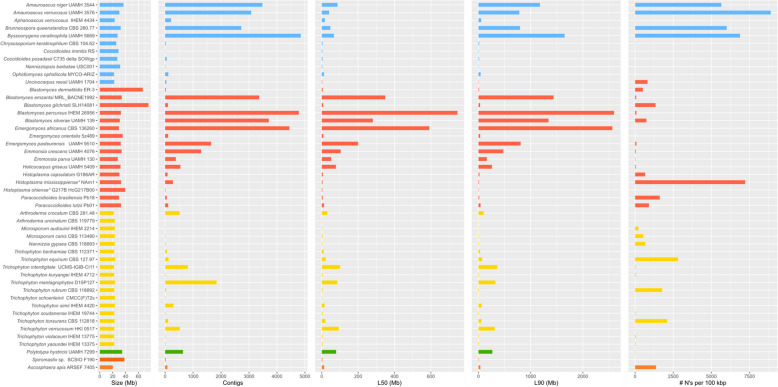
Genome quality statistics for reference genomes of the order Onygenales available in the NCBI database. Color coding indicates taxonomic families: blue, Onygenaceae; red, Ajellomycetaceae; yellow, Arthrodermataceae; green, Incertae sedis; brown, Spiromastigoidaceae; orange, Ascosphaeraceae. *nom. inval

To identify and remove potential contaminating sequences, we created a custom Kraken 2 database comprising high-quality genomes phylogenetically close to the contaminated genomes. This custom database, called “CustomDB” was generated, to improve the contaminated Onygenaceae genomes. To create the CustomDB, the remaining reference genomes belonging to the Onygenales available in the NCBI database were first downloaded (https://www.ncbi.nlm.nih.gov/datasets/genome/?taxon=33183&reference_only=true; accessed in April 2023) (The full list of genomes is shown in Supplementary Table 1). Subsequently, quality and completeness analyses were then performed for each genome using QUAST and BUSCO for each genome (Fig. 2). In addition, the genomes were analyzed with Kraken 2 and the StandardDB database as described above. The genomes with the highest quality, i.e., lowest percentage of contamination, less than 500 contigs, and more than 95% Complete BUSCOs, were selected to become part of the CustomDB (Fig. 1B).

To minimize the inclusion of potential contaminants in the CustomDB, the high-quality Onygenales genomes were first screened with the Standard Kraken 2 database (StandardDB). Sequences classified as contaminants were extracted, validated by BLAST, Then mapped back to the original genomes with Bowtie2 v2.2.5 [39], and masked using Samtools v0.1.20 [40] and BEDtools v2.30.0. [41]. After masking, the cleaned genomes were added to the Kraken 2 library, the “CustomDB” database was built using the kraken2-build option.

Finally, to eliminate possible contaminating sequences from previously identified putatively contaminated Onygenaceae genomes, these assemblies were analyzed using CustomDB with the *–classified-out* option (Fig. 1C).

### Assessment of the decontamination methodology

The efficacy of the decontamination methodology was evaluated by comparing putatively contaminated genomes with their corresponding filtered version. Post-decontamination quality was assessed using QUAST, BUSCO, and Kraken 2 (with the StandardDB) as previously described. Both genome versions (putatively contaminated and filtered) were then annotated using BRAKER2 v2.1.6 pipeline [42], incorporating GeneMark-ES and AUGUSTUS packages. Functional annotation was performed using InterProScan [43], focusing on Pfam domain analysis to assess functional differences between genome versions (Fig. 1C).

Additionally, a benchmark set of high-quality Onygenales genomes was used for comparative evaluation. This subset included protein data from two strains from the family Onygenaceae (*Chrysosporium keratinophilum* CBS 104.62 and *Coccidioides immitis* RS), one strain from the family Ajellomycetaceae (*Histoplasma capsulatum* G186AR), and one from the family Arthrodermataceae (*Trichophyton* [*Tr.*] *benhamiae* CBS 112371). Additionally, *Aspergillus nidulans* FGSC A4 (order Eurotiales, family Aspergillaceae) was included as an outgroup to the Onygenales set. Its phylogenetic distance from Onygenales makes it a suitable reference for evaluating domain composition and annotation patterns. Protein sequences were downloaded from NCBI GenBank (https://www.ncbi.nlm.nih.gov/genome/?term=Onygenaceae).

To visualize the presence of domains associated with bacteria, eukaryotes, and shared domains, in both the putatively contaminated and filtered genomes, as well as in the high-quality benchmark genomes, a heatmap was generated using the Pheatmap R library [44] (Fig. 4).

### Genome indexes and phylogenetic analysis

To compare the genetic relatedness between the putatively contaminated genomes and the filtered genomes, Average Nucleotide Identity (ANI) was calculated using the Orthologous Average Nucleotide Identity Tool (OrthoANI) v0.93.1 implemented in the OAT software [45] with default settings. Comparisons were made using the most closely related genomes of the Onygenaceae family, i.e. *C. posadasii* (C735 delta SOWgp), *C. immitis* (RS), *Ap. verrucosus* (IHEM 4434), *Ch. keratinophilum* (CBS 104.62), and *U. reesii* (UAMH 1704).

For the phylogenetic analysis, protein sequences of *C. posadasii* (C735 delta SOWgp), *C. immitis* (RS), and *U. reesii* (UAMH 1704) were downloaded, while the genomes of *Ap. verrucosus* (IHEM 4434) and *Ch. keratinophilum* (CBS 104.62) were annotated using the Braker2. Orthologous groups among the analyzed strains were predicted using Orthofinder v2.5.5 [46] with default settings. Single-copy orthologous sequences (longer than 200 amino acids) were aligned with MAFFT v7.5 [47], and poorly or ambiguously aligned positions were trimmed using Gblocks v0.91b [48]. The resulting alignments were concatenated into a super-alignment. The best protein substitution model was determined using ModelTest-NG v0.1.7 [49]. Finally, Maximum Likelihood phylogenetic analysis was performed using IQ-TREE v2.2.0.3 [50] with the JTT + I + G4 + F model and 1000 ultrafast bootstrap replicates [51].

## Results

### Quality and completeness of the original Onygenaceae genomes

The Onygenaceae genomes revealed a total length between 21.97 and 36.71 Mbp and a G + C content between 40.36% and 53.15%. QUAST quality analysis revealed two distinct groups. One group exhibited a high count of contigs (> 1,000), > 1,000 N’s per 100 kbp, and modest N50 values (< 300,000). This group included *Am. niger* UAMH 3544, *Am. verrucosus* UAMH 3576, *Br. queenslandica* CBS 280.77 and *By. ceratinophila* UAMH 5669. These genomes were sequenced using the Illumina platform and assembled with the SOAPdenovo pipeline. In contrast, the other group displayed a low count of contigs (< 300), approximately 20 N’s per 100 kbp, and higher values for N50 and N90 (Table 1); notably, although all four genomes with fragmented assemblies shared the same sequencing technology and assembler, one genome classified as high-quality (*Ap. verrucosus*) was also sequenced using Illumina and assembled with SOAPdenovo. This suggests that sequencing platform and assembly software alone do not fully explain the observed fragmentation.

**Fig. 3:**
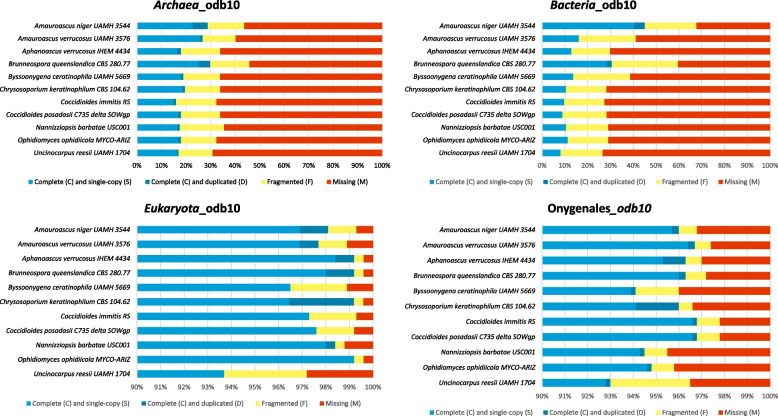
BUSCO completeness scores for Onygenaceae reference genomes using four lineage datasets: Archaea_odb10, Bacteria_odb10, Eukaryota_odb10, and Onygenales_odb10. Each bar is subdivided by BUSCO category: light blue, complete and single-copy genes; dark blue, complete and duplicated genes; yellow, fragmented genes; red, missing genes

The "*Onygenales*_odb10" lineage was automatically selected by BUSCO for all Onygenaceae genomes. The percentage of complete BUSCOs ranged from 93.0% to 96.8%, while fragmented BUSCOs ranged from 0.8% to 1.9%. Although relatively low percentages (< 20%) of bacterial and archaeal BUSCOs were detected across most genomes, likely reflecting conserved domains or spurious hits, the genomes of *Am. niger* UAMH 3544 and *Br. queenslandica* CBS 280.77 displayed notably higher proportions of complete BUSCOS for Bacteria (“*Bacteria*_odb10”) and Archaea ("*Archaea*_odb10"), with values of 45.1% and 30.6% for Bacteria, and 29.9% and 28.9% for Archaea, respectively (Table 1 and Fig. 3).

### Contamination identification and removal

Kraken 2 highlighted a higher percentage of bacterial sequences in the genomes of *Am. niger* UAMH 3544, *Am. verrucosus* UAMH 3576, *Br. queenslandica* CBS 280.77, and *By. ceratinophila* UAMH 5669, accounting for 11.27%, 7.07%, 12.05%, and 4.98%, respectively. For Eukarya, the genomes displayed values below 2.25%. It is important to note that the total percentage of Eukarya was associated with *Homo sapiens*, in contrast to other domains, where the total percentage was the sum of the different species associated with the domain. Contamination levels for Archaea and viruses were less than 0.10% and 0.04%, respectively (Table 1). The four most contaminated genomes consistently harbored the same five main contaminants, identified as *Acidovorax*, *Ramlibacter*, *Sphingomonas*, *Variovorax,* and *Homo* (indicative of potential human DNA contamination). To confirm Kraken 2 classifications, sequences assigned to these taxa were extracted and validated via BLAST against the NCBI nt database, reinforcing their identification as contaminants. Notably, for the sequences classified as *Homo* by Kraken 2, BLAST reported, “No significant similarity found”. Based on quality statistics and contamination levels, the genomes of *Am. niger* UAMH 3544, *Am. verrucosus* UAMH 3576, *Br. queenslandica* CBS 280.77, and *By. ceratinophila* UAMH 5669 were selected for improvement.

### Database creation

Based on the quality assessment of the Onygenales genomes (completeness, contiguity, and degree of contamination) (Fig. 2, Supplementary Table 1), *Arthroderma* [*Ar.*] *uncinatum* CBS 119779 (GCA_011692745.1)*, Ap. verrucosus* IHEM 4434 (GCA_014839905.1)*, Ch. keratinophilum* CBS 104.62 (GCA_029850275.1)*, Na. gypsea* CBS 118893 (GCA_000150975.2)*,* and *Tr. benhamiae* CBS 112371 (GCA_000151125.2) were included in the CustomDB database (Supplementary Table 2).

The previously selected genomes with highly fragmented assemblies (*Am. niger* UAMH 3544, *Am. verrucosus* UAMH 3576, *Br. queenslandica* CBS 280.77, and *By. ceratinophila* UAMH 5669) were used as input files for Kraken2 with CustomDB. The results of the comparisons between the genomes prior to and after analysis are shown in Table 2.

**Table 2:** Comparison of statistics of selected Onygenaceae genomes before and after the analysis

Species (Strain)		*Amauroascus niger* (UAMH 3544)		*Amauroascus verrucosus* (UAMH 3576)		*Brunneospora queenslandica* (CBS 280.77)		*Byssoonygena ceratinophila* (UAMH 5669)	
		Before	After	Before	After	Before	After	Before	After
Assembly statistics									
**# Contigs**		3,481	330	3,075	185	2,724	190	4,851	553
**Total length (bp)**		36,718,296	24,756,779	30,376,016	23,353,341	32,335,957	23,009,572	27,454,949	21,444,050
**Largest contig (bp)**		564,389	564,389	836,833	836,833	979,930	979,930	643,678	643,678
**G + C content (%)**		50.09	47.58	49.26	46.91	53.15	49.41	48.44	47.88
**N50 (bp)**		98,932	175,671	217,754	321,426	173,791	260,216	103,459	150,193
**N90 (bp)**		3,899	46,458	3,183	78,520	4,367	81,216	1,404	18,782
**L50**		86	42	40	26	47	26	67	43
**L90**		1,178	143	784	81	798	81	1,651	177
**# N's per 100 kbp**		5,647.86	204.06	8,888.54	65.73	6,001.89	111.44	6,870.54	57.07
BUSCO statistics									
***Onygenales*_odb10**	C* (%)	96.0	95.2	96.7	96.6	96.3	96.3	94.1	92.5
	F* (%)	0.8	0.8	0.7	0.7	0.9	0.8	1.9	1.5
	M* (%)	3.2	4.0	2.6	2.7	2.8	2.9	4.0	6.0
	**n**	4,862	4,862	4,862	4,862	4,862	4,862	4,862	4,862
***Archaea*_odb10**		C:28.9%,n:194	C:18.5%,n:194	C:19.6%,n:194	C:18.5%,n:194	C:29.9%,n:194	C:19.0%,n:194	C:19.0%,n:194	C:18.5%,n:194
***Bacteria*_odb10**		C:45.1%,n:124	C:9.7%,n:124	C:16.1%,n:124	C:11.3%,n:124	C:30.6%,n:124	C:9.7%,n:124	C:13.7%,n:124	C:11.3%,n:124
***Eukaryota*_odb10**		C:98.1%,n:255	C:96.5%,n:255	C:97.7%,n:255	C:97.7%,n:255	C:99.2%,n:255	C:99.2%,n:255	C:96.5%,n:255	C:94.1%,n:255
Percentage of fragments covered for each domain in the analyzed genomes obtained by Kraken 2 with StandardDB									
**Viruses**		0.02	0.01	0.01	0.01	0.01	0.01	0.02	0.01
**Archaea**		0.06	0.03	0.04	0.03	0.07	0.04	0.04	0.04
**Bacteria**		11.27	1.19	7.07	1.04	12.05	1.21	4.98	1.23
**Eukaryota**		1.12	1.43	0.83	1.07	0.41	0.55	0.58	0.66
** Total**		12.47	2.66	7.95	2.15	12.54	1.80	5.62	1.95
Number of genes and Pfam domains identified									
**Number of genes**		11,427	7,534	8,646	6,688	10,658	6,875	8,598	6,613
**Pfam domains**		17,92	9,718	12,053	9,271	17,266	9,347	10,546	8,838

C*, Complete BUSCOs; F*, Fragmented BUSCOs; M*, Missing BUSCOs; n, Total BUSCO groups searched

### Quality and completeness of the decontaminated Onygenaceae genomes 

The outcomes of the QUAST analysis demonstrated that filtering with CustomDB reduced the number of contigs, total genome length, and number of ambiguous bases (N’s per 100 kbp) in all decontaminated genomes (Table 2). Additionally, a modest decrease in GC content (1–3%) and improvements in N50 and N90 metrics were observed. Importantly, the largest contig size remained consistent between filtered and unfiltered genomes in all cases (Table 2).

The BUSCO results showed a consistent decrease in Complete BUSCOs associated with Archaea and Bacteria lineages in all genomes, confirming the targeted removal of likely contaminant regions. The largest reduction was observed in *Am. niger* UAMH 3544, from 28.9% to 18.5% (20 genes) in the *Archaea*_odb10 lineage, and from 45.1% to 9.7% (44 genes) in the *Bacteria*_odb10 lineage. Conversely, in the *Eukaryota*_odb10 and *Onygenales*_odb10 lineages, a minor decrease in the number of Complete BUSCOs was noted. The most marked reduction was in *By. ceratinophila* UAMH 5669, decreasing from 96.5% to 94.1% (6 genes) in the *Eukaryota*_odb10 lineage, and from 94.1% to 92.5% (73 genes) in the *Onygenales*_odb10 lineage (Table 2). This suggests that some fungal sequences were likely removed during the filtering process. We acknowledge this trade-off and emphasize that while BUSCO completeness slightly decreased, overall contamination levels, particularly from bacterial and archaeal sources, were significantly reduced, supporting the effectiveness of our decontamination approach.

The filtered genomes showed a reduction in contamination according to Kraken 2 analysis, with levels falling below 2.66% in all cases. The greatest reduction occurred in the Bacteria, followed by Archaea and viruses. Notably, despite the overall decrease, Eukarya showed a slight increase, most prominently a 0.31% rise in *Am. niger* UAMH 3544, though this remains well below the original contamination levels (Table 2).

### Functional annotation and genome indexes of the decontaminated Onygenaceae genomes

**Fig. 4:**
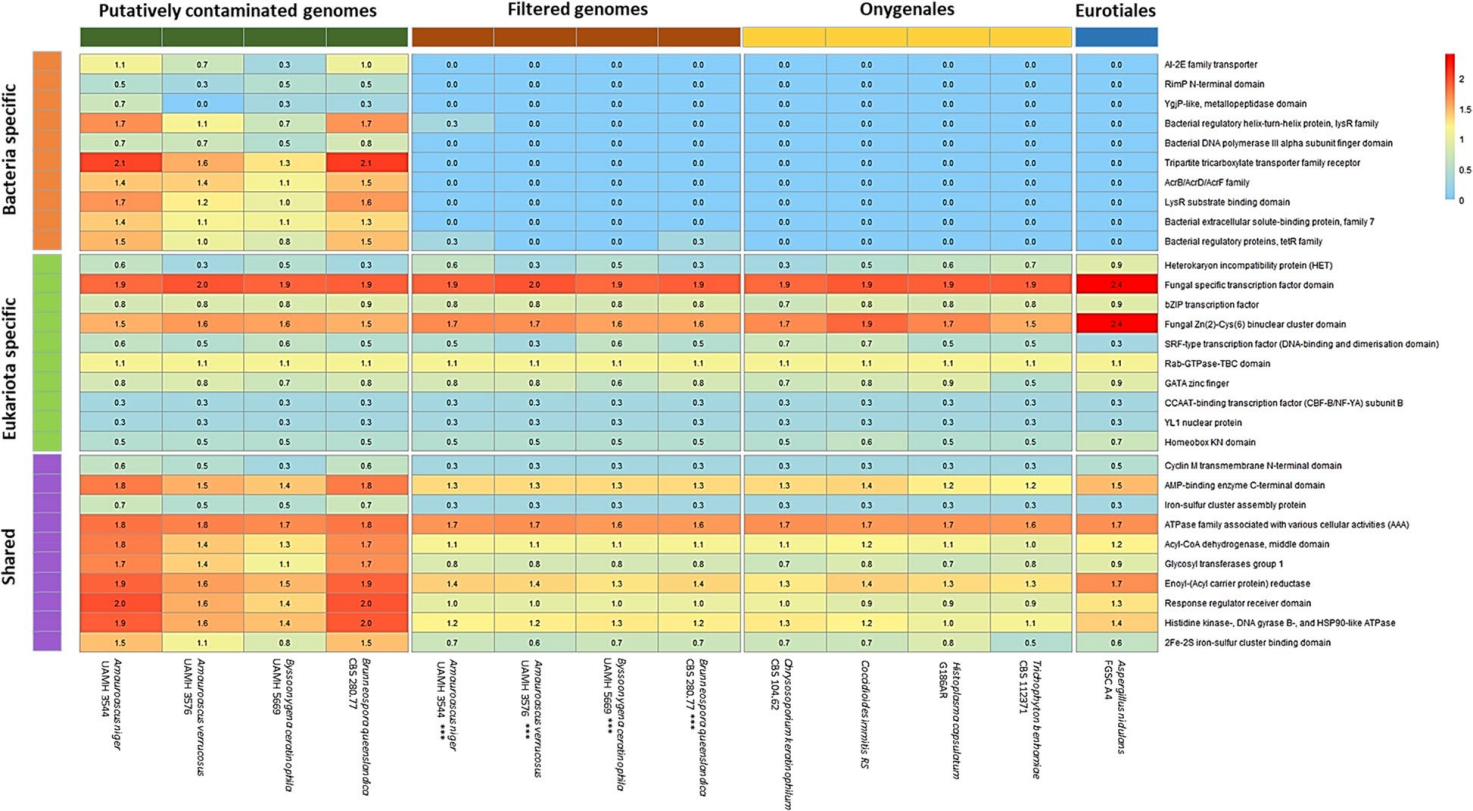
Heatmap of Pfam domain abundances across four genome categories. (1) Putatively contaminated genomes: selected Onygenaceae genomes prior to contamination filtering; (2) Filtered genomes: Onygenaceae genomes after contaminant removal; (3) Onygenales; high-quality reference genomes from other closely related Onygenales species; (4) Eurotiales: genome of *Aspergillus nidulans* FGSC A4, used as an outgroup. Pfam domains are grouped into three categories: Bacteria-specific (predominantly found in bacteria), Eukaryota-specific, and Shared (present in both domains). The heatmap displays log-transformed domain counts using the formula log(n + 1) to stabilize variance and reduce the influence of highly abundant features

The annotation and Pfam analysis revealed a consistent reduction in the number of gene predictions and annotations in the filtered genomes. The largest reduction was observed in *Br. queenslandica* CBS 280.77, with gene count declining from 10,658 to 6,875, and Pfam domains from 17,266 to 9,347. In contrast, the smallest reduction was observed in *Am. verrucosus* UAMH 3576, which decreased from 8,646 to 6,688. *By. ceratinophila* UAMH 5669 exhibited the smallest decrease in Pfam domains, from 10,546 to 8,838 (Table 2). Pfam analysis revealed a large reduction in the number of Pfam domains previously associated with Bacteria and Archaea. This encompassed a wide range of functions, including regulatory proteins, protein receptors, and components of the secretion system, which were consistently observed in the filtered genomes. In addition, a decrease in the number of Pfam domains shared between prokaryotes and eukaryotes was observed (Fig. 4).

In contrast, fungal-specific domains remained stable before and after filtering, comparable to those in high-quality Onygenales genomes. The filtered genomes also showed a consistent reduction in Pfam domains shared between prokaryotes and eukaryotes. The filtered values were close to or identical to those of the other high-quality genomes analyzed (Fig. 4).

**Fig. 5:**
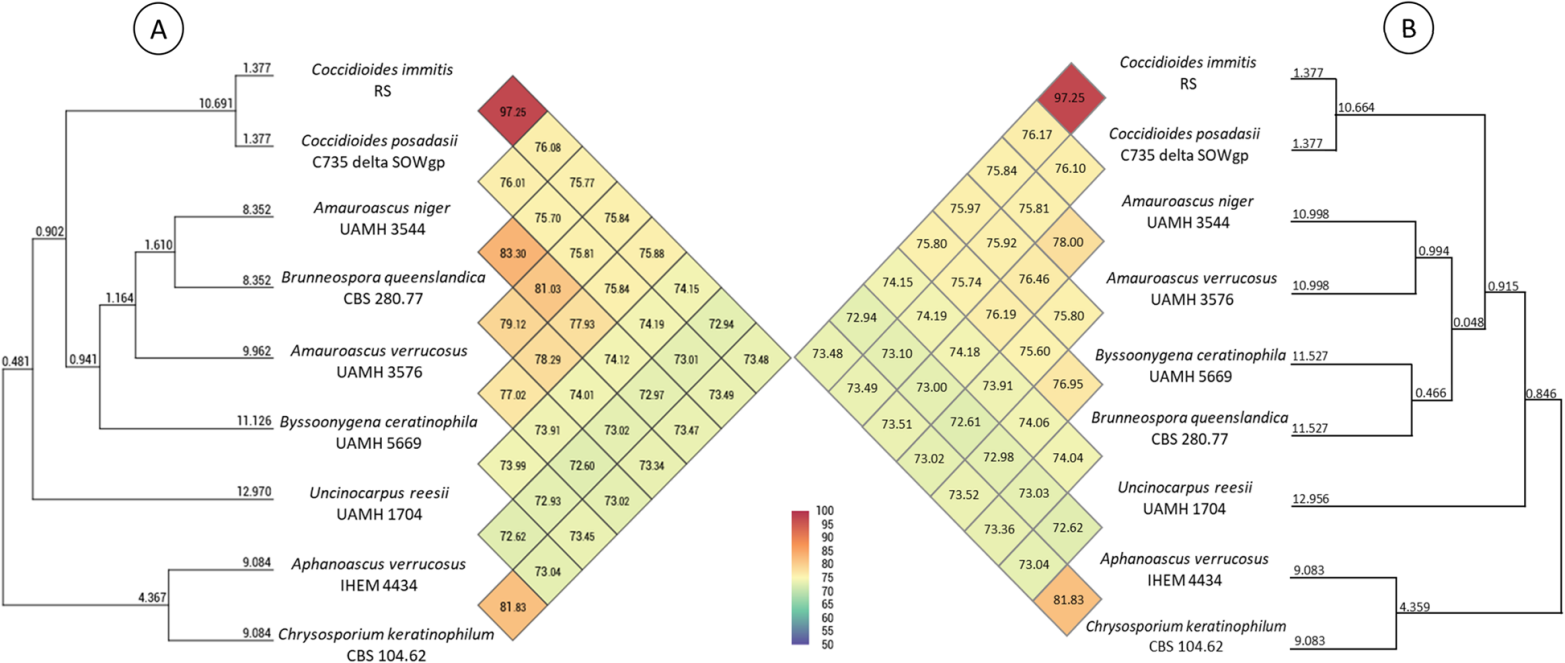
Heatmap and dendrogram of Average Nucleotide Identity (ANI) among Onygenaceae genomes. **A** ANI analysis performed on the original genomes. **B** ANI analysis performed on the filtered genomes. Pairwise ANI values were computed using the OrthoANI tool, which also generated the hierarchical clustering dendrogram using the UPGMA algorithm

Changes in ANI values were observed between the test with the original genomes and the filtered genomes. In all cases, ANI values increased when comparing two filtered genomes, from 1.22 between *Am. niger* (UAMH 3544) and *By. ceratinophila* (UAMH 5669) to 7.11 between *Br. queenslandica* (CBS 280.77) and *Am. niger* (UAMH 3544). Conversely, when comparing one filtered genome against other Onygenaceae genomes, only minor increases (< 0.1) were observed. No changes were detected in ANI values for the remaining Onygenaceae genomes (Figs. 5A, B).

The UPGMA dendrograms derived from ANI analysis of the original genomes and the filtered genomes showed slight topological differences. In the original dataset (Fig. 5A), *Am. niger* UAMH 3544 and *Br. queenslandica* CBS 280.77 were grouped in the same clade, while *Am. verrucosus* UAMH 3576 and *By. ceratinophila* UAMH 5669 were placed in two separate but related branches. However, in the dendrogram constructed based on the filtered genomes (Fig. 5B), *Am. niger* UAMH 3544 and *Am. verrucosus* UAMH 3576 grouped together, and *By. ceratinophila* UAMH 5669 clustered with *Br. queenslandica* CBS 280.77 (Fig. 5B). While these shifts suggest changes in overall genomic similarity following contaminant removal, it is important to note that UPGMA is a simple distance-based clustering method and does not provide robust phylogenetic inference. To more accurately assess evolutionary relationships, we constructed a maximum likelihood (ML) phylogenetic tree based on conserved single-copy orthologs.

The orthofinder results using the filtered genomes showed a decrease in the total number of proteins identified (from 79,119 to 67,500) and in the number of proteins assigned to orthogroups (from 74,698 to 64,534) relative to the original genomes. Conversely, the number of orthogroups present in all species increased (from 4,176 to 4,343) as did the number of single-copy orthogroups (from 3,520 to 3,765). The concatenated alignment matrix also increase in total characters, from 1,949,624 to 2,056,428. Importantly, the phylogenetic trees inferred from both analyzes exhibited the same topology.

The maximum likelihood (ML) phylogenetic tree showed four strongly supported clades (100/100 ultra-fast bootstrap support). The first clade included *Am. niger* UAMH 3544 and *Am. verrucosus* UAMH 3576. The second clade included *By. ceratinophila* UAMH 5669 and *Br. queenslandica* CBS 280.77. The third clade included *C. immitis* RS and *C. posadasii* C735 delta SOWgp. In a separate branch was *U. reesii* (UAMH 170), while *Ap. verrucosus* (IHEM 4434) and *Ch. keratinophilum* (CBS 104.62) were grouped in another clade far from the rest (Supplementary Fig. 1).

## Discussion

In recent years, the number of fungal genomes available in public databases has increased exponentially, providing valuable insights into medicine, agriculture, taxonomy, biotechnology, and ecology [52–56]. However, as previous studies have shown, these genomic datasets often include sequences that do not belong to the target organism. Contaminating sequences can undermine the accuracy of downstream analyses, leading to inflated genome sizes, misannotation, erroneous gene counts, and spurious phylogenetic inferences. Therefore, we assessed and improved the quality of four publicly available fungal genomes from the family Onygenaceae. Our approach used closely related genomes of high quality, such as *Ar. uncinatum* CBS 119779*, Ap. verrucosus* IHEM 4434*, Ch. keratinophilum* CBS 104.62*, Na. gypsea* CBS 118893*,* and* Tr. benhamiae* CBS 112371, to reduce apparent contamination based on k-mer classification and mitigate the associated risk and inaccuracies of using these genomes.

Although basic assembly metrics such as genome size, G + C content, contig count, N50, and genome completeness [57] provide useful quality indicators, they are insufficient to confirm or rule out the presence of contamination. Unusual values within these metrics, however, may raise suspicion of potential contamination that requires further investigation [31, 58]. Some authors have proposed minimum quality standards for genome publication, submission, and reuse; however, most focus primarily on prokaryotic organisms [59–61]. Eukaryotic genomes pose additional challenges due to their larger size, higher repeat content, horizontal gene transfer, and lineage-specific genomic features.

Several tools, including Kraken2, EukCC, ContScout, and FCS-GX, have been developed to better address these complexities of eukaryotic genomes [15, 17, 20, 32]. Cornet and Baurain [21] recently benchmarked some of these tools and found that Kraken 2 performs particularly well at detecting cross-domain contamination between eukaryotes and prokaryotes. Despite their high sensitivity and specificity, such tools may also yield false positives or negatives, so they require complementary approaches and manual curation [20].

Contamination in publicly available datasets is not a marginal issue. In 2020, over 2.1 million, 114,035, and 14,148 contaminant sequences were identified in RefSeq, GenBank, and NR, respectively. In 2022, it was estimated that approximately one in three eukaryotic genomes at NCBI contained contaminating sequences, and in 2024, 36.8 Gbp of contamination were identified among 1.6 million assemblies, predominantly in eukaryotic genomes. This vulnerability is attributed to the larger genome size and high repeat content of eukaryotes, which complicate assembly and error detection [20, 32, 62].

To date, most contamination analyses have focused on studying a broad diversity of organisms. Consequently, like other taxa, fungi are typically analyzed at high taxonomic levels (e.g., kingdom or phylum). FCS-GX analyses revealed distinct contamination patterns across fungal lineages: Ascomycota genomes were predominantly contaminated with γ-proteobacteria, Basidiomycota with high-GC Gram-positive bacteria, and budding yeasts with Basidiomycota sequences. In contrast, ContScout identified bacterial contaminants as the major contaminant group across Ascomycota, Basidiomycota, and Mucoromycota. However, genus-level characterizations of these bacterial contaminants remains limited [20, 32, 62].

In our analysis, when assessed exclusively for completeness using the Bowers et al. classification framework [59], all examined genomes would be classified as “high-quality drafts”. However, based on Kraken 2 contamination analysis, the genomes of *Am. niger* UAMH 3544 and *Br. queenslandica* CBS 280.77 exceeded 10% contamination and thus qualify as 'low-quality drafts', while the genomes of *Am. verrucosus* UAMH 3576, *By. ceratinophila* UAMH 5669 and *Na. barbatae* USC001 fell into the ‘medium-quality drafts’ category. This underscores the limitations of completeness-based metrics alone.

Contamination in sequencing data arises from both intrinsic and extrinsic factors. Intrinsic sources include endogenous symbionts, sample type and origin, and cultivation conditions. Extrinsic factors encompass cross-contamination between samples, variations in DNA extraction and sequencing protocols, reagent contaminants, and bioinformatic artifacts such as chimeric assemblies or low-coverage regions [8, 10, 30, 63]. Consistent with prior reports, we detected frequent contaminants in the analyzed genomes, suggesting that some of these could be systemic issues rather than isolated incidents.

For example, sequences from *Sphingomonas* bacteria were among the prevalent contaminants detected; this bacterial genus has been previously reported in ultrapure water systems, laboratory reagents, and negative controls (template-free "blank" DNA extractions) [10, 64, 65]. Other bacterial genera such as *Acidovorax* and *Variovorax* have also been reported as common contaminants in sequencing plates and control samples [66, 67].

Notably, we also detected *Ramlibacter*, which has not been previously documented as a contaminant in sequencing projects. Its presence was confirmed both by Kraken 2 and BLASTn alignment against the NCBI nt database and corroborated by the NCBI Foreign Contamination Screen (FCS), which similarly flagged *Ramlibacter* sequences in the fungal genomes (Supplementary Table 3). The consistent detection of these taxa across all four genomes suggests a potential shared contamination source. However, due to the lack of sequencing metadata and negative controls in the analyzed datasets, we cannot conclusively determine whether this reflects a systemic laboratory contaminant or an isolated artifact introduced during the generation of these specific datasets [68].

Regarding *Homo sapiens*, sequences are well-known contaminants in genome and protein databases [22, 23, 62], In our case, Kraken 2 flagged some sequences as “*Homo*”, but BLASTn, searches showed “No significant similarity found”, despite originating from genomes deposited in NCBI. This discrepancy likely stems from differences in classification approaches [17, 69]. Specifically, the standard Kakern2 database includes only human genome among eukaryotes, so k-mers from other eukaryotic repetitive sequences may be misclassified as human due to similarity. In contrast, BLASTn filters out repetitive or low-complexity sequences and short, non-significant hits. Therefore, human-associated contamination was ruled out in the analyzed genomes.

Finally, we observed some cases where Kraken 2 detected low-level contamination (< 2%), while the FCS-GX tool did not flag these sequences (Table 1). Subsequent BLASTn searches again returned “No significant similarity found”. This apparent contradiction may be explained by methodological differences among tools, including their sensitivity, specificity, and detection thresholds, as well as the fact that BLAST filters out short, repetitive, or statistically non-significant matches.

Following contaminant removal, we observed substantial reductions in both size and contig number. These findings align with previous studies demonstrating that contaminant sequences are primarily located in small contigs [23, 26, 62]. This occurs because contaminant sequences are incompatible with the target organism’s genome, which complicates assembly processes. As Breitwieser et al. [23], demonstrated, such sequences typically exhibit low coverage, promoting their incorporation into smaller contigs. However, contig size alone is not a reliable indicator of contamination, as fragmentation may also result from the inherent complexity of the genome.

Importantly, removal of contaminants increased the proportion of reads classified as Eukaryota. This likely reflects both a relative decrease in non-Eukaryota reads and reclassification of ambiguous sequences after cleaning. The contaminating sequences we identified are unlikely to stem from horizontal gene transfer or mobile elements, given their distribution, identification, and consistency. Nonetheless, these genomes were sequenced in 2015 − 2016, when data analysis and contamination-cleaning tools were not as advanced as they are today.

We additionally compared the detection of potential cross-domain contamination using BUSCO’s Auto-Select Lineage and Kraken 2. Our results indicate that BUSCO can serve as a preliminary indicator of severe cross-domain contamination, consistent with previous studies suggesting that this feature can signal serious contamination problems between taxonomic domains [58, 70]. However, BUSCO was not originally designed for contamination detection, and should be interpreted cautiously, particularly in eukaryotic genomes such as fungi, where gene loss, horizontal gene transfer, and endosymbiotic associations may confound the analysis.

The impact of contaminants extends beyond basic assembly metrics. Contaminants can inflate genome size, fragment assemblies, mislead gene annotation, and lead to predictions of nonexistent or chimeric genes, skewing pathway inference and gene copy number estimation. This was exemplified in the human genome project, where initial predictions of 30,000–40,000 protein-coding genes were later corrected to 20,000 after removal of bacterial contaminants [23, 71–74]. Similarly, in our comparative Pfam analysis, contaminant removal revealed numerous foreign PFAM domains unrelated to the target organisms. These spurious domains could lead to misannotation of genes, genetic elements, or functional domains. Such errors may introduce discrepancies between computational predictions and experimental validations or inconsistencies when replicating results across different research groups [23, 28].

The Average Nucleotide Identity (ANI) has been widely used to validate genomic variation and improve taxonomic assignments in archaeal and bacterial genomes, making it possible to establish cut-off values to differentiate species and genera [75–78]. This is due to its ability to generate data that correlate closely with the results of the DNA-DNA hybridization (DDH) method, the “gold standard” method for the classification of prokaryotes [77, 79–81]. However, in fungi, this tool has only been applied to a limited number of taxa [82–85]. In the present study, the original genomes showed higher ANI values than the filtered genomes, which could be explained by the presence of ubiquitous contaminants in these genomes, as shown by the results of Kraken 2, which increases the number of similar regions and thus the ANI values. However, we acknowledge that this hypothesis is not definitively demonstrated here, as we did not perform alignment-based confirmation or phylogenetic inconsistency checks to trace the source of these shifts. While similar phenomena have been observed in prokaryotic genome studies [77], caution is warranted when extrapolating such findings to eukaryotes, given their more complex genome architecture and evolutionary dynamics. These results highlight the impact of contaminating sequences on the accuracy of rapid taxonomic verification methods such as ANI.

Previous studies have highlighted the effects of contaminating sequences on the topology and bootstrap values of resulting phylogenetic trees [86–88]. However, in our study, phylogenetic analysis showed no differences between the obtained phylogenies or bootstrap support. This can be attributed to the fact that the phylogenetic analysis was based on conserved, single-copy orthologous genes present in all genomes. Consequently, any contaminating sequence would have to be consistently present in all genomes analyzed, making it unlikely to affect the total number of single-copy orthologs shared by the organisms. Notably, the dendrogram derived from the ANI analysis of the filtered genomes closely matched the phylogenetic tree based on single-copy orthologs, further demonstrating the efficiency of our strategy.

Compared with previous reports that have addressed genome contamination at broader taxonomic levels, typically spanning kingdoms or phyla, our study provided a genus- and family-level framework that enabled a more precise and context-sensitive detection of contaminants. This represents a key advance over earlier works, which often focused on prokaryotic genomes or presented only global estimates of contamination in eukaryotic datasets. The detection of contaminants such as *Ramlibacter*, which had not been previously documented in sequencing projects, illustrates the sensitivity of our approach and its potential to reveal overlooked or novel sources of contamination.

Building on these findings, we aim to extend this work to encompass a wider diversity of fungi, including species of medical and biotechnological relevance. Such an expansion will not only strengthen the reliability of basic research but also enhance applications in clinical diagnostics, pathogen surveillance, and industrial biotechnology. The flexibility and scalability of our pipeline make it applicable not only to other fungal groups but also to broader taxonomic datasets available in repositories such as NCBI. Furthermore, integrating contamination screening with detailed metadata curation will help clarify the origins of recurrent contaminants, enable the differentiation between systemic and sample-specific artifacts, and ultimately contribute to the development of cleaner and more reliable genomic resources.

Finally, it is important to emphasize that this methodology, which is based on sequence similarity between genomes, may be prone to excluding genes originating from recent horizontal transfer events. Because such genes, if absent in the ancestor of the species used to construct the database, are unlikely to exhibit sufficient similarity and may be inadvertently omitted.

## Conclusions

Our research highlights that many fungal genomes from the Onygenaceae family, as available in public databases, may contain substantial levels of contamination, predominantly from bacterial sources. This underscores the critical need for rigorous quality control of genomic data prior to use to prevent errors or misinterpretation in scientific studies.

By utilizing a curated database of high-quality genomes closely related to the target species, we were able to identify and remove putative contaminant sequences, leading to improvements in basic assembly metrics and functional annotation consistency. However, we recognize that these results are preliminary and that more organisms and transcriptomic data are required for validation. Additionally, it remains possible that some legitimate lineage-specific or horizontally transferred genes were inadvertently removed.

Overall, this study underscores the value of implementing systematic quality control pipelines in fungal genomics, while also emphasizing the need for refinement and validation of contamination detection strategies in eukaryotic genomes.

## Considerations

The effectiveness of contaminant sequence removal depends directly on the quality of the genomes included in the custom database. If the database lacks closely related genomes or includes low-quality genomes, there is a risk that some contaminant sequences may remain undetected, or, conversely, that authentic sequences from the target genome could be erroneously removed. This concern is particularly relevant for sequences arising from recent horizontal gene transfers, lineage-specific expansions, or rapidly evolving regions that may not have close homologs in the reference set.

While our approach enables the identification of likely contaminants using taxonomic classification, it relies on the assumption that reference genomes sufficiently capture the diversity of the target taxon. This introduces the possibility of systematically excluding genuine biological variation that diverges from the database-defined "core".

Therefore, although a customized reference database can improve detection of well-characterized contaminants, it should be applied with caution. Lastly, the processing of each genome requires a thorough preliminary assessment to determine whether the inclusion of a customized database enhances genome quality or introduces additional errors.

## Supplementary Information

Supplementary Material 1.

## Data Availability

The genome sequences analyzed in this study are listed in Supplementary Table 1, which includes the GenBank assembly accessions available at the National Center for Biotechnology Information (NCBI) database (https://www.ncbi.nlm.nih.gov/). The four decontaminated genomes generated during the present study, along the custom database, are available at Zenodo (https://zenodo.org/records/14073432) and have also been deposited in DDBJ/ENA/GenBank under the BioSample accessions SAMN51891636, SAMN51891637, SAMN51891638, and SAMN51891639.
