# Supplementary material for: Contamination of fungal genomes of Onygenaceae (Phylum Ascomycota) in public databases: incidence, detection, and impact
Source: BMC Genomics. 2025 Nov 19;26:1057. doi: 10.1186/s12864-025-12223-3 (PMC12628603; doi:10.1186/s12864-025-12223-3)
Supplement: Supplementary file 1 — Supplementary Material 1. [file 12864_2025_12223_MOESM1_ESM.pptx]

## Slide 1
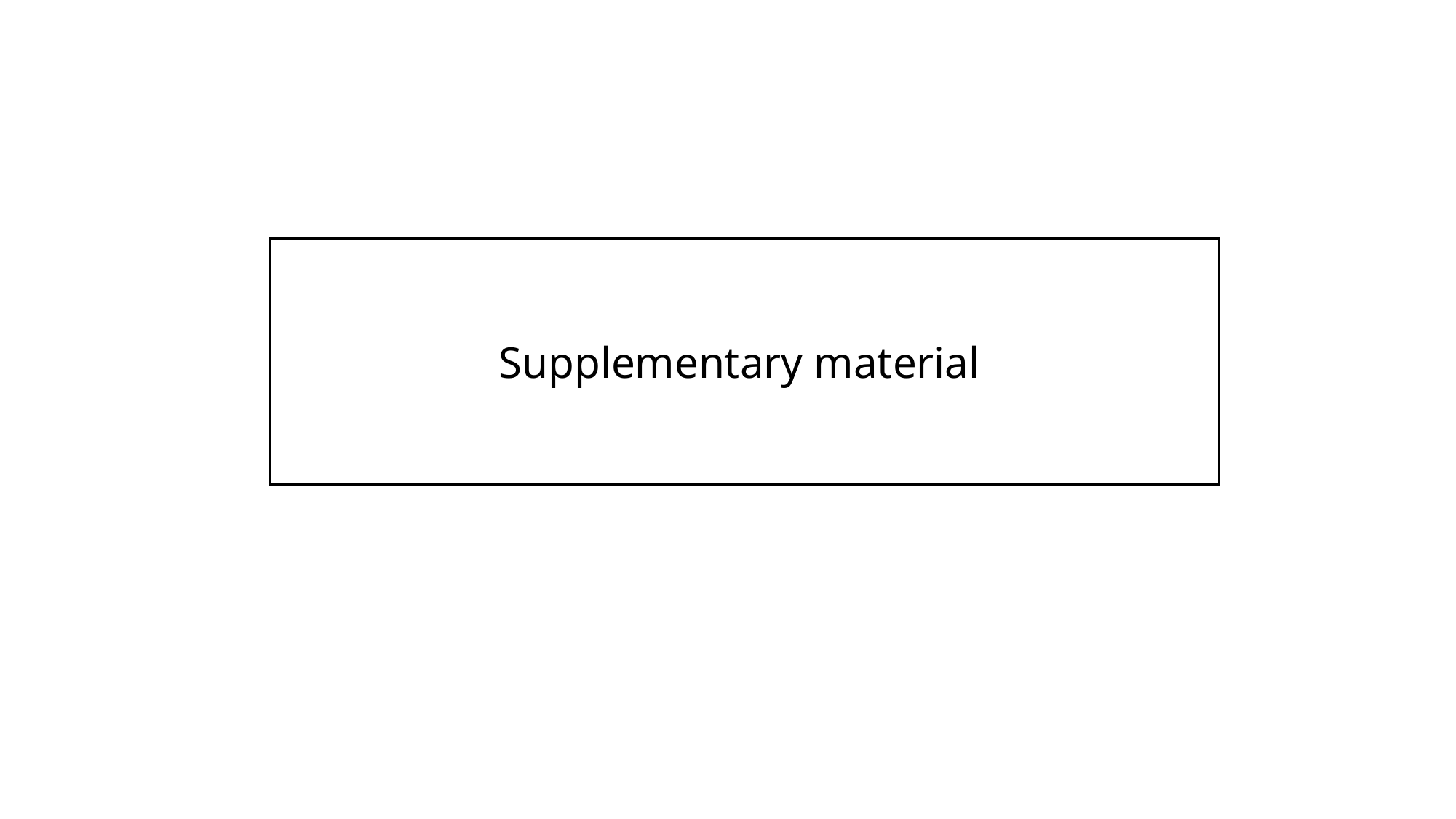

Supplementary material

## Slide 2
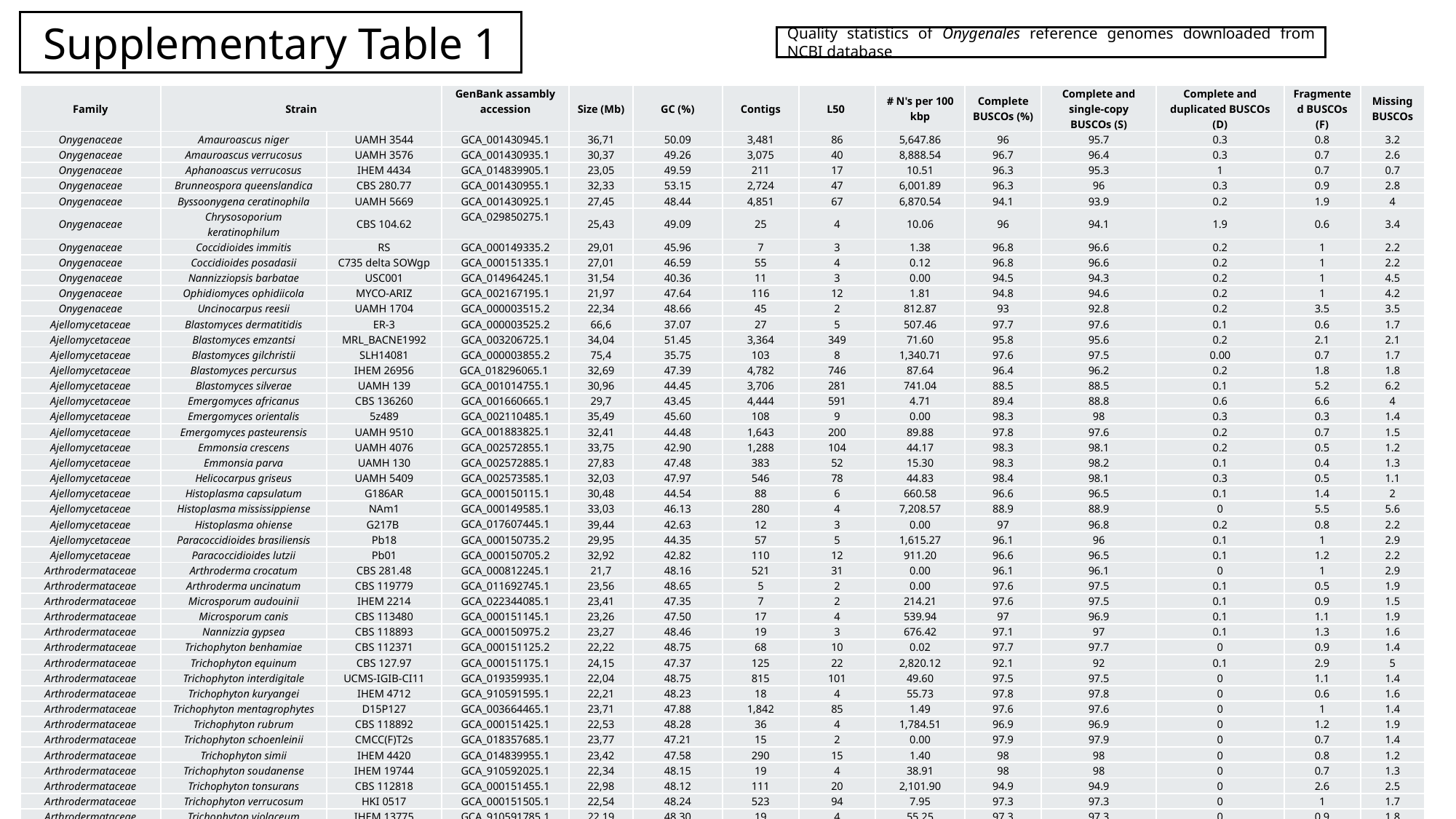

Supplementary Table 1
Quality statistics of Onygenales reference genomes downloaded from NCBI database
| Family | Strain | | GenBank assambly accession | Size (Mb) | GC (%) | Contigs | L50 | # N's per 100 kbp | Complete BUSCOs (%) | Complete and single-copy BUSCOs (S) | Complete and duplicated BUSCOs (D) | Fragmented BUSCOs (F) | Missing BUSCOs |
| --- | --- | --- | --- | --- | --- | --- | --- | --- | --- | --- | --- | --- | --- |
| Onygenaceae | Amauroascus niger | UAMH 3544 | GCA\_001430945.1 | 36,71 | 50.09 | 3,481 | 86 | 5,647.86 | 96 | 95.7 | 0.3 | 0.8 | 3.2 |
| Onygenaceae | Amauroascus verrucosus | UAMH 3576 | GCA\_001430935.1 | 30,37 | 49.26 | 3,075 | 40 | 8,888.54 | 96.7 | 96.4 | 0.3 | 0.7 | 2.6 |
| Onygenaceae | Aphanoascus verrucosus | IHEM 4434 | GCA\_014839905.1 | 23,05 | 49.59 | 211 | 17 | 10.51 | 96.3 | 95.3 | 1 | 0.7 | 0.7 |
| Onygenaceae | Brunneospora queenslandica | CBS 280.77 | GCA\_001430955.1 | 32,33 | 53.15 | 2,724 | 47 | 6,001.89 | 96.3 | 96 | 0.3 | 0.9 | 2.8 |
| Onygenaceae | Byssoonygena ceratinophila | UAMH 5669 | GCA\_001430925.1 | 27,45 | 48.44 | 4,851 | 67 | 6,870.54 | 94.1 | 93.9 | 0.2 | 1.9 | 4 |
| Onygenaceae | Chrysosoporium keratinophilum | CBS 104.62 | GCA\_029850275.1 | 25,43 | 49.09 | 25 | 4 | 10.06 | 96 | 94.1 | 1.9 | 0.6 | 3.4 |
| Onygenaceae | Coccidioides immitis | RS | GCA\_000149335.2 | 29,01 | 45.96 | 7 | 3 | 1.38 | 96.8 | 96.6 | 0.2 | 1 | 2.2 |
| Onygenaceae | Coccidioides posadasii | C735 delta SOWgp | GCA\_000151335.1 | 27,01 | 46.59 | 55 | 4 | 0.12 | 96.8 | 96.6 | 0.2 | 1 | 2.2 |
| Onygenaceae | Nannizziopsis barbatae | USC001 | GCA\_014964245.1 | 31,54 | 40.36 | 11 | 3 | 0.00 | 94.5 | 94.3 | 0.2 | 1 | 4.5 |
| Onygenaceae | Ophidiomyces ophidiicola | MYCO-ARIZ | GCA\_002167195.1 | 21,97 | 47.64 | 116 | 12 | 1.81 | 94.8 | 94.6 | 0.2 | 1 | 4.2 |
| Onygenaceae | Uncinocarpus reesii | UAMH 1704 | GCA\_000003515.2 | 22,34 | 48.66 | 45 | 2 | 812.87 | 93 | 92.8 | 0.2 | 3.5 | 3.5 |
| Ajellomycetaceae | Blastomyces dermatitidis | ER-3 | GCA\_000003525.2 | 66,6 | 37.07 | 27 | 5 | 507.46 | 97.7 | 97.6 | 0.1 | 0.6 | 1.7 |
| Ajellomycetaceae | Blastomyces emzantsi | MRL\_BACNE1992 | GCA\_003206725.1 | 34,04 | 51.45 | 3,364 | 349 | 71.60 | 95.8 | 95.6 | 0.2 | 2.1 | 2.1 |
| Ajellomycetaceae | Blastomyces gilchristii | SLH14081 | GCA\_000003855.2 | 75,4 | 35.75 | 103 | 8 | 1,340.71 | 97.6 | 97.5 | 0.00 | 0.7 | 1.7 |
| Ajellomycetaceae | Blastomyces percursus | IHEM 26956 | GCA\_018296065.1 | 32,69 | 47.39 | 4,782 | 746 | 87.64 | 96.4 | 96.2 | 0.2 | 1.8 | 1.8 |
| Ajellomycetaceae | Blastomyces silverae | UAMH 139 | GCA\_001014755.1 | 30,96 | 44.45 | 3,706 | 281 | 741.04 | 88.5 | 88.5 | 0.1 | 5.2 | 6.2 |
| Ajellomycetaceae | Emergomyces africanus | CBS 136260 | GCA\_001660665.1 | 29,7 | 43.45 | 4,444 | 591 | 4.71 | 89.4 | 88.8 | 0.6 | 6.6 | 4 |
| Ajellomycetaceae | Emergomyces orientalis | 5z489 | GCA\_002110485.1 | 35,49 | 45.60 | 108 | 9 | 0.00 | 98.3 | 98 | 0.3 | 0.3 | 1.4 |
| Ajellomycetaceae | Emergomyces pasteurensis | UAMH 9510 | GCA\_001883825.1 | 32,41 | 44.48 | 1,643 | 200 | 89.88 | 97.8 | 97.6 | 0.2 | 0.7 | 1.5 |
| Ajellomycetaceae | Emmonsia crescens | UAMH 4076 | GCA\_002572855.1 | 33,75 | 42.90 | 1,288 | 104 | 44.17 | 98.3 | 98.1 | 0.2 | 0.5 | 1.2 |
| Ajellomycetaceae | Emmonsia parva | UAMH 130 | GCA\_002572885.1 | 27,83 | 47.48 | 383 | 52 | 15.30 | 98.3 | 98.2 | 0.1 | 0.4 | 1.3 |
| Ajellomycetaceae | Helicocarpus griseus | UAMH 5409 | GCA\_002573585.1 | 32,03 | 47.97 | 546 | 78 | 44.83 | 98.4 | 98.1 | 0.3 | 0.5 | 1.1 |
| Ajellomycetaceae | Histoplasma capsulatum | G186AR | GCA\_000150115.1 | 30,48 | 44.54 | 88 | 6 | 660.58 | 96.6 | 96.5 | 0.1 | 1.4 | 2 |
| Ajellomycetaceae | Histoplasma mississippiense | NAm1 | GCA\_000149585.1 | 33,03 | 46.13 | 280 | 4 | 7,208.57 | 88.9 | 88.9 | 0 | 5.5 | 5.6 |
| Ajellomycetaceae | Histoplasma ohiense | G217B | GCA\_017607445.1 | 39,44 | 42.63 | 12 | 3 | 0.00 | 97 | 96.8 | 0.2 | 0.8 | 2.2 |
| Ajellomycetaceae | Paracoccidioides brasiliensis | Pb18 | GCA\_000150735.2 | 29,95 | 44.35 | 57 | 5 | 1,615.27 | 96.1 | 96 | 0.1 | 1 | 2.9 |
| Ajellomycetaceae | Paracoccidioides lutzii | Pb01 | GCA\_000150705.2 | 32,92 | 42.82 | 110 | 12 | 911.20 | 96.6 | 96.5 | 0.1 | 1.2 | 2.2 |
| Arthrodermataceae | Arthroderma crocatum | CBS 281.48 | GCA\_000812245.1 | 21,7 | 48.16 | 521 | 31 | 0.00 | 96.1 | 96.1 | 0 | 1 | 2.9 |
| Arthrodermataceae | Arthroderma uncinatum | CBS 119779 | GCA\_011692745.1 | 23,56 | 48.65 | 5 | 2 | 0.00 | 97.6 | 97.5 | 0.1 | 0.5 | 1.9 |
| Arthrodermataceae | Microsporum audouinii | IHEM 2214 | GCA\_022344085.1 | 23,41 | 47.35 | 7 | 2 | 214.21 | 97.6 | 97.5 | 0.1 | 0.9 | 1.5 |
| Arthrodermataceae | Microsporum canis | CBS 113480 | GCA\_000151145.1 | 23,26 | 47.50 | 17 | 4 | 539.94 | 97 | 96.9 | 0.1 | 1.1 | 1.9 |
| Arthrodermataceae | Nannizzia gypsea | CBS 118893 | GCA\_000150975.2 | 23,27 | 48.46 | 19 | 3 | 676.42 | 97.1 | 97 | 0.1 | 1.3 | 1.6 |
| Arthrodermataceae | Trichophyton benhamiae | CBS 112371 | GCA\_000151125.2 | 22,22 | 48.75 | 68 | 10 | 0.02 | 97.7 | 97.7 | 0 | 0.9 | 1.4 |
| Arthrodermataceae | Trichophyton equinum | CBS 127.97 | GCA\_000151175.1 | 24,15 | 47.37 | 125 | 22 | 2,820.12 | 92.1 | 92 | 0.1 | 2.9 | 5 |
| Arthrodermataceae | Trichophyton interdigitale | UCMS-IGIB-CI11 | GCA\_019359935.1 | 22,04 | 48.75 | 815 | 101 | 49.60 | 97.5 | 97.5 | 0 | 1.1 | 1.4 |
| Arthrodermataceae | Trichophyton kuryangei | IHEM 4712 | GCA\_910591595.1 | 22,21 | 48.23 | 18 | 4 | 55.73 | 97.8 | 97.8 | 0 | 0.6 | 1.6 |
| Arthrodermataceae | Trichophyton mentagrophytes | D15P127 | GCA\_003664465.1 | 23,71 | 47.88 | 1,842 | 85 | 1.49 | 97.6 | 97.6 | 0 | 1 | 1.4 |
| Arthrodermataceae | Trichophyton rubrum | CBS 118892 | GCA\_000151425.1 | 22,53 | 48.28 | 36 | 4 | 1,784.51 | 96.9 | 96.9 | 0 | 1.2 | 1.9 |
| Arthrodermataceae | Trichophyton schoenleinii | CMCC(F)T2s | GCA\_018357685.1 | 23,77 | 47.21 | 15 | 2 | 0.00 | 97.9 | 97.9 | 0 | 0.7 | 1.4 |
| Arthrodermataceae | Trichophyton simii | IHEM 4420 | GCA\_014839955.1 | 23,42 | 47.58 | 290 | 15 | 1.40 | 98 | 98 | 0 | 0.8 | 1.2 |
| Arthrodermataceae | Trichophyton soudanense | IHEM 19744 | GCA\_910592025.1 | 22,34 | 48.15 | 19 | 4 | 38.91 | 98 | 98 | 0 | 0.7 | 1.3 |
| Arthrodermataceae | Trichophyton tonsurans | CBS 112818 | GCA\_000151455.1 | 22,98 | 48.12 | 111 | 20 | 2,101.90 | 94.9 | 94.9 | 0 | 2.6 | 2.5 |
| Arthrodermataceae | Trichophyton verrucosum | HKI 0517 | GCA\_000151505.1 | 22,54 | 48.24 | 523 | 94 | 7.95 | 97.3 | 97.3 | 0 | 1 | 1.7 |
| Arthrodermataceae | Trichophyton violaceum | IHEM 13775 | GCA\_910591785.1 | 22,19 | 48.30 | 19 | 4 | 55.25 | 97.3 | 97.3 | 0 | 0.9 | 1.8 |
| Arthrodermataceae | Trichophyton yaoundei | IHEM 13375 | GCA\_910592095.1 | 22,36 | 48.11 | 17 | 4 | 40.91 | 97.4 | 97.4 | 0 | 1 | 1.6 |
| Ascosphaeraceae | Ascosphaera apis | ARSEF 7405 | GCA\_001636715.1 | 20,31 | 47.66 | 82 | 13 | 1,369.07 | 81.5 | 81.3 | 0.2 | 5.1 | 13.4 |
| Spiromastigoidaceae | Spiromastix sp. | SCSIO F190 | GCA\_014805645.1 | 38,12 | 44.75 | 22 | 3 | 0.00 | 95.9 | 95.3 | 0.6 | 0.8 | 3.3 |
| Incertae sedis | Polytolypa hystricis | UAMH 7299 | GCA\_002573605.1 | 34,69 | 45.63 | 641 | 79 | 11.26 | 97.3 | 97.2 | 0.1 | 0.9 | 1.8 |

## Slide 3
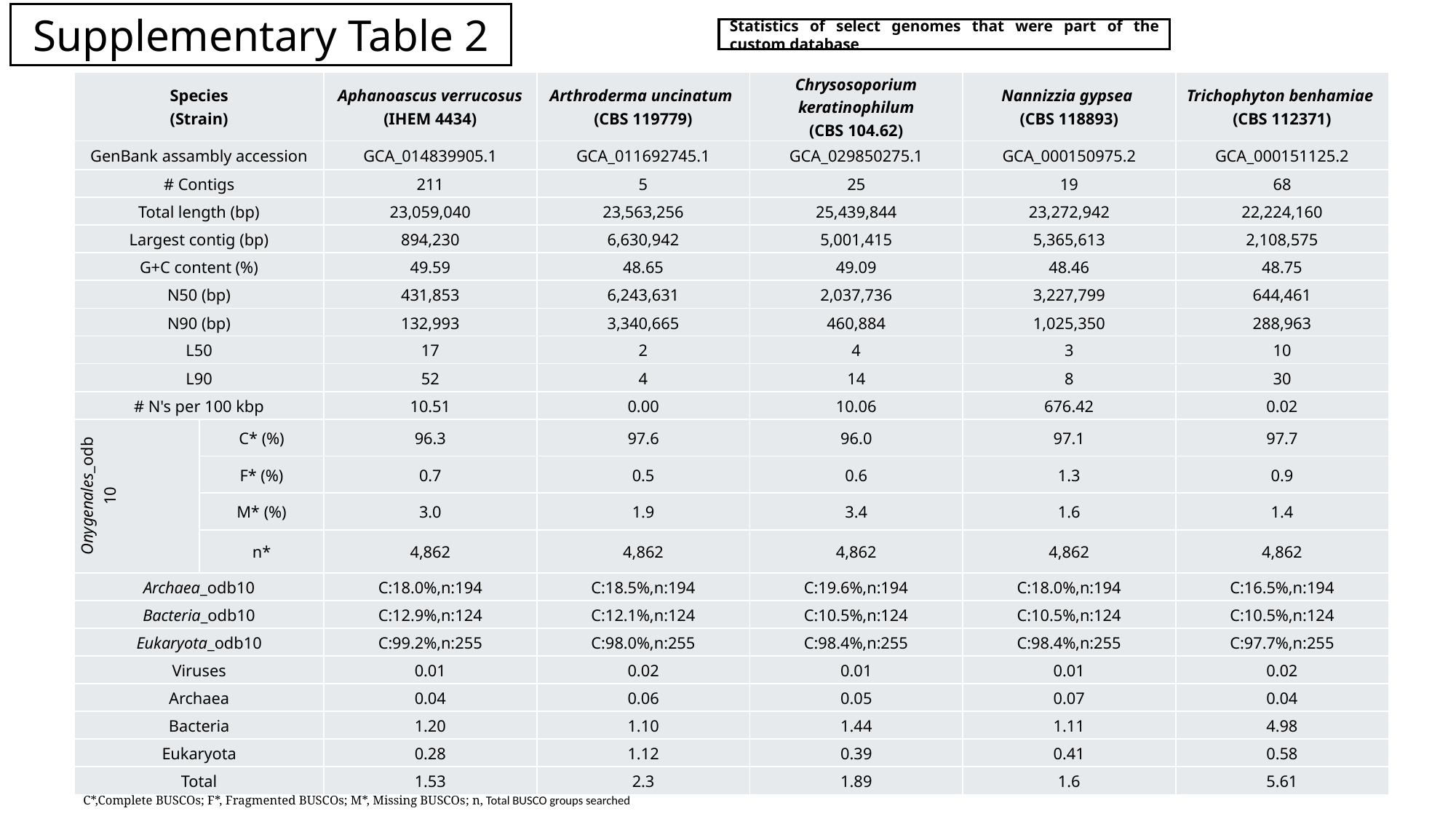

Supplementary Table 2
Statistics of select genomes that were part of the custom database
| Species (Strain) | | Aphanoascus verrucosus (IHEM 4434) | Arthroderma uncinatum (CBS 119779) | Chrysosoporium keratinophilum (CBS 104.62) | Nannizzia gypsea (CBS 118893) | Trichophyton benhamiae (CBS 112371) |
| --- | --- | --- | --- | --- | --- | --- |
| GenBank assambly accession | | GCA\_014839905.1 | GCA\_011692745.1 | GCA\_029850275.1 | GCA\_000150975.2 | GCA\_000151125.2 |
| # Contigs | | 211 | 5 | 25 | 19 | 68 |
| Total length (bp) | | 23,059,040 | 23,563,256 | 25,439,844 | 23,272,942 | 22,224,160 |
| Largest contig (bp) | | 894,230 | 6,630,942 | 5,001,415 | 5,365,613 | 2,108,575 |
| G+C content (%) | | 49.59 | 48.65 | 49.09 | 48.46 | 48.75 |
| N50 (bp) | | 431,853 | 6,243,631 | 2,037,736 | 3,227,799 | 644,461 |
| N90 (bp) | | 132,993 | 3,340,665 | 460,884 | 1,025,350 | 288,963 |
| L50 | | 17 | 2 | 4 | 3 | 10 |
| L90 | | 52 | 4 | 14 | 8 | 30 |
| # N's per 100 kbp | | 10.51 | 0.00 | 10.06 | 676.42 | 0.02 |
| Onygenales\_odb10 | C\* (%) | 96.3 | 97.6 | 96.0 | 97.1 | 97.7 |
| | F\* (%) | 0.7 | 0.5 | 0.6 | 1.3 | 0.9 |
| | M\* (%) | 3.0 | 1.9 | 3.4 | 1.6 | 1.4 |
| | n\* | 4,862 | 4,862 | 4,862 | 4,862 | 4,862 |
| Archaea\_odb10 | | C:18.0%,n:194 | C:18.5%,n:194 | C:19.6%,n:194 | C:18.0%,n:194 | C:16.5%,n:194 |
| Bacteria\_odb10 | | C:12.9%,n:124 | C:12.1%,n:124 | C:10.5%,n:124 | C:10.5%,n:124 | C:10.5%,n:124 |
| Eukaryota\_odb10 | | C:99.2%,n:255 | C:98.0%,n:255 | C:98.4%,n:255 | C:98.4%,n:255 | C:97.7%,n:255 |
| Viruses | | 0.01 | 0.02 | 0.01 | 0.01 | 0.02 |
| Archaea | | 0.04 | 0.06 | 0.05 | 0.07 | 0.04 |
| Bacteria | | 1.20 | 1.10 | 1.44 | 1.11 | 4.98 |
| Eukaryota | | 0.28 | 1.12 | 0.39 | 0.41 | 0.58 |
| Total | | 1.53 | 2.3 | 1.89 | 1.6 | 5.61 |
C*,Complete BUSCOs; F*, Fragmented BUSCOs; M*, Missing BUSCOs; n, Total BUSCO groups searched

## Slide 4
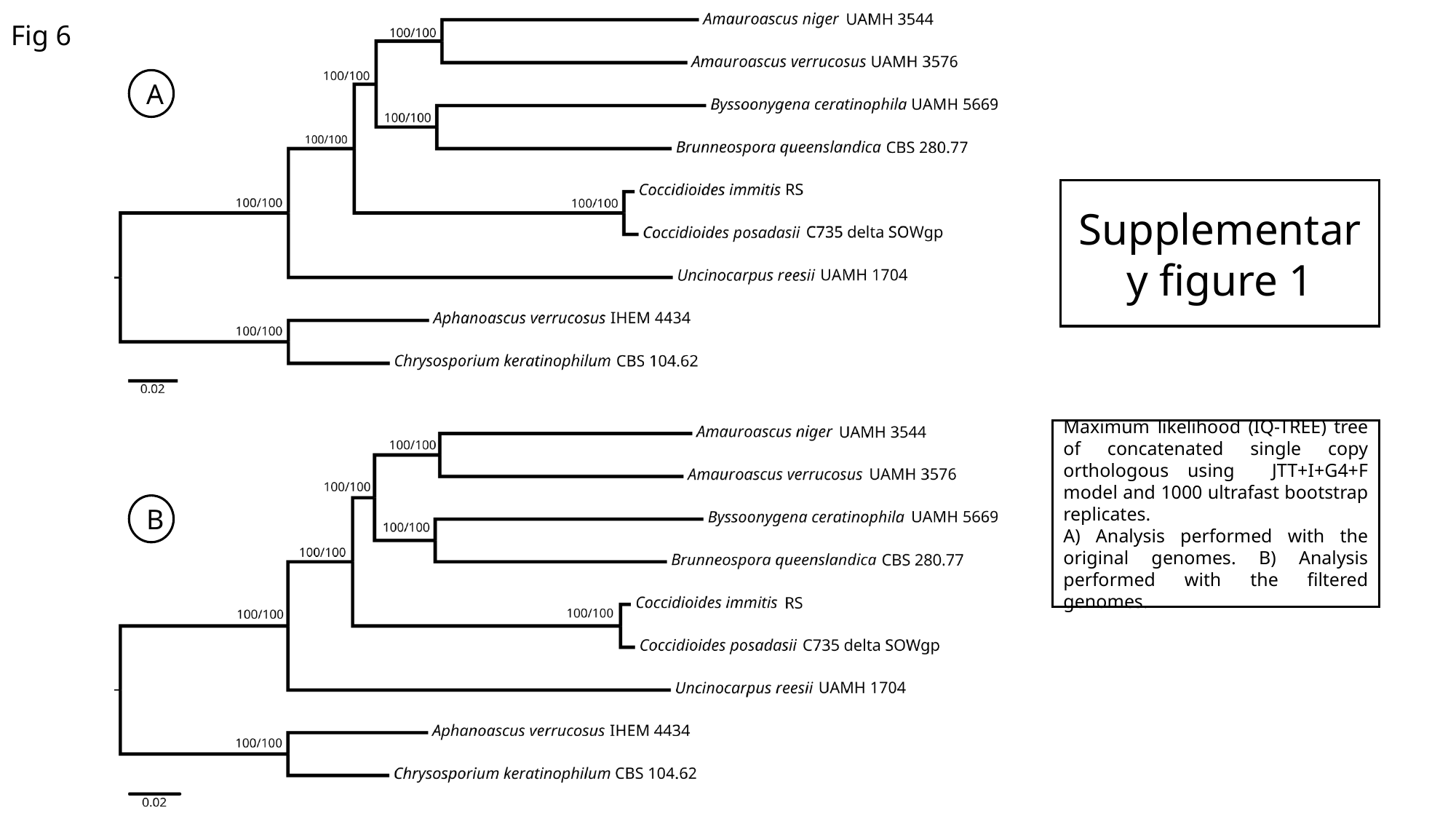

Fig 6
A
Supplementary figure 1
Maximum likelihood (IQ-TREE) tree of concatenated single copy orthologous using JTT+I+G4+F model and 1000 ultrafast bootstrap replicates.
A) Analysis performed with the original genomes. B) Analysis performed with the filtered genomes.
B

## Slide 5
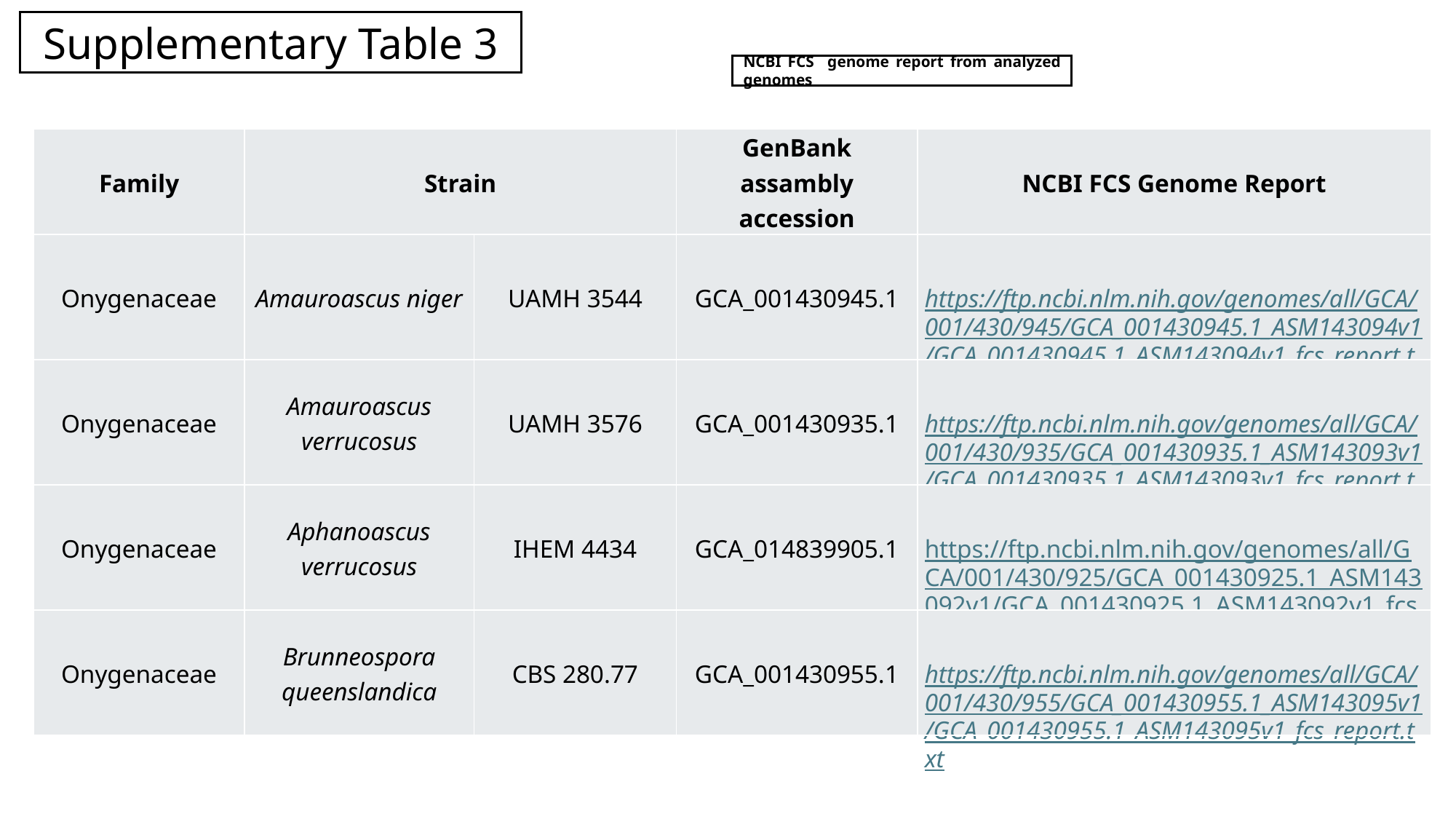

Supplementary Table 3
NCBI FCS genome report from analyzed genomes
| Family | Strain | | GenBank assambly accession | NCBI FCS Genome Report |
| --- | --- | --- | --- | --- |
| Onygenaceae | Amauroascus niger | UAMH 3544 | GCA\_001430945.1 | https://ftp.ncbi.nlm.nih.gov/genomes/all/GCA/001/430/945/GCA\_001430945.1\_ASM143094v1/GCA\_001430945.1\_ASM143094v1\_fcs\_report.txt |
| Onygenaceae | Amauroascus verrucosus | UAMH 3576 | GCA\_001430935.1 | https://ftp.ncbi.nlm.nih.gov/genomes/all/GCA/001/430/935/GCA\_001430935.1\_ASM143093v1/GCA\_001430935.1\_ASM143093v1\_fcs\_report.txt |
| Onygenaceae | Aphanoascus verrucosus | IHEM 4434 | GCA\_014839905.1 | https://ftp.ncbi.nlm.nih.gov/genomes/all/GCA/001/430/925/GCA\_001430925.1\_ASM143092v1/GCA\_001430925.1\_ASM143092v1\_fcs\_report.txt |
| Onygenaceae | Brunneospora queenslandica | CBS 280.77 | GCA\_001430955.1 | https://ftp.ncbi.nlm.nih.gov/genomes/all/GCA/001/430/955/GCA\_001430955.1\_ASM143095v1/GCA\_001430955.1\_ASM143095v1\_fcs\_report.txt |
